# The Unexplored Virome of Two Atlantic Coast Fish: Contribution of Next-Generation Sequencing to Fish Virology

**DOI:** 10.3390/foods9111634

**Published:** 2020-11-09

**Authors:** Andreia Filipa-Silva, Ricardo Parreira, Sandra Martínez-Puchol, Sílvia Bofill-Mas, Maria Teresa Barreto Crespo, Mónica Nunes

**Affiliations:** 1 ITQB NOVA, Instituto de Tecnologia Química e Biológica António Xavier, Universidade Nova de Lisboa, Av. da República, 2780-157 Oeiras, Portugal; andreiasilva@ibet.pt (A.F.-S.); tcrespo@ibet.pt (M.T.B.C.); 2 iBET, Instituto de Biologia Experimental e Tecnológica, Apartado 12, 2781-901 Oeiras, Portugal; 3 Global Health and Tropical Medicine (GHTM) Research Center, Unidade de Microbiologia Médica, Instituto de Higiene e Medicina Tropical (IHTM), Universidade Nova de Lisboa (NOVA), 1349-008 Lisboa, Portugal; ricardo@ihmt.unl.pt; 4 Laboratory of Viruses Contaminants of Water and Food, Genetics, Microbiology & Statistics Department, Universitat de Barcelona, 08028 Barcelona, Catalonia, Spain; smpuchol@gmail.com (S.M.-P.); sbofill@ub.edu (S.B.-M.); 5 The Water Research Institute (idRA), Universitat de Barcelona, 08001 Barcelona, Catalonia, Spain

**Keywords:** virome diversity, metagenomics, Atlantic horse mackerel, gilthead seabream, health-threat

## Abstract

Much of the knowledge on viruses is focused on those that can be propagated using cell-cultures or that can cause disease in humans or in economically important animals and plants. However, this only reflects a small portion of the virosphere. Therefore, in this study, we explore by targeted next-generation sequencing, how the virome varies between Atlantic horse mackerels and gilthead seabreams from fisheries and aquaculture from the center and south regions of Portugal. Viral genomes potentially pathogenic to fish and crustaceans, as well as to humans, were identified namely *Astroviridae*, *Nodaviridae*, *Hepadnaviridae*, *Birnaviridae*, *Caliciviridae*, and *Picornaviridae* families. Also bacteriophages sequences were identified corresponding to the majority of sequences detected, with *Myoviridae*, *Podoviridae*, and *Siphoviridae*, the most widespread families in both fish species. However, these findings can also be due to the presence of bacteria in fish tissues, or even to contamination. Overall, seabreams harbored viruses from a smaller number of families in comparison with mackerels. Therefore, the obtained data show that fish sold for consumption can harbor a high diversity of viruses, many of which are unknown, reflecting the overall uncharacterized virome of fish. While cross-species transmission of *bonafide* fish viruses to humans is unlikely, the finding of human pathogenic viruses in fish suggest that fish virome can be a potential threat regarding food safety.

## 1. Introduction

As an important source of food and revenue for millions of people globally, it was estimated that fish production in 2018 reached 179 million tons, 87 percent of which were used directly for human consumption [1]. While the demand for fish may be rising, viral diseases are a major factor constraining fish trade and aquaculture production, affecting the development of fish industry with substantial economic losses worldwide [2,3]. Furthermore, the increasing demand for seafood with the subsequent growth of aquaculture systems, and the impact of climate change, offer new opportunities for the transmission of both novel and previously characterized pathogens, namely viruses [3].

Marine environments are known for their viral diversity and richness. Bacteriophages, commonly found in marine and freshwater systems, play an important role in the aquatic environments balance as predators of bacteria, representing one of the most abundant organisms in fish [4,5,6]. In addition to the presence of bacteriophages, recent studies suggest that fish harbor a greater number of viruses than any other class of vertebrates, including multiple families of RNA viruses previously thought to infect only mammals [3,7]. For instance, the discovery of hepadnaviruses and filoviruses in fish implies that these viruses have ancient vertebrate origins, and that their evolution possibly required more host jumping than previously realized, involving a possible move from aquatic to terrestrial vertebrates [3,7,8].

Valuable insights into virus ecology and evolution are provided when using metagenomics approaches [3,7], with benefits for aquaculture [3] and food security. For instance, information about outbreaks associated with human pathogenic viruses in fish is scarce. However, the Centers for Disease Control and Prevention (CDC) compiles searchable lists of viral outbreaks that can be retrieved from the National Outbreak Reporting System [9]. In this system, regarding the years between 2008 and 2018, fish alone were responsible for 14 noroviruses (NoV) outbreaks, totaling 177 human infections and 3 hospitalizations.

Much of our present knowledge of fish viruses is focused either on those that act as agents of disease for economically relevant species, or those that can be easily isolated in cell culture, representing a small proportion of the overall viral diversity [10]. To circumvent this problem, metagenomic approaches provide the possibility for an in-depth characterization of the molecular diversity of viruses present in a range of environments, potentially revealing the entire virus composition associated with an individual or *taxa*—i.e., its virome—even when they are not associated with evident disease [7,11,12]. Metagenomic-based studies and characterization of more viruses can also offer important new data about evolutionary processes, as well the factors that may mediate differences in virus composition between different species of fish, and the frequency with which viruses jump over species borders [3]. Moreover, it can provide insightful information on how viral community composition changes, which depends on regional and local processes, including interactions occurring between fish, as well as environmental factors such as temperature, salinity, or dissolved oxygen, that determine fish distribution [13,14]. Virome diversity in fish populations is also affected by how viral transmission between fish occurs: primarily through feces, contaminated water and/or broodstocks, culture of multiple fish species in close proximity, or the use of unsterilized fish products (e.g., feed) in aquaculture productions [15].

Therefore, in this study, the diversity of viruses associated with two of the most highly captured, farmed and consumed fish species in Europe was evaluated using a metagenomic approach. We aimed to determine: (i) how virus composition varies between species; (ii) how virus composition varies between fisheries from different regions and aquaculture within the same species; (iii) whether a large and dense host population is associated with a greater number of viruses when compared to a more solitary counterpart; (iv) and, if the analyzed fish virome could pose a risk regarding food safety. To address these goals, targeted next-generation sequencing (NGS) was used to evaluate the virome of wild gilthead seabreams (*Sparus aurata*) and of Atlantic horse mackerels (*Trachurus trachurus*) from fisheries of Portugal, namely from Peniche and Algarve regions, as well as farmed gilthead seabreams from Algarve. 

## 2. Materials and Methods

### 2.1. Fish Sample Collection and Nucleic Acids Extraction

**Figure 1:**
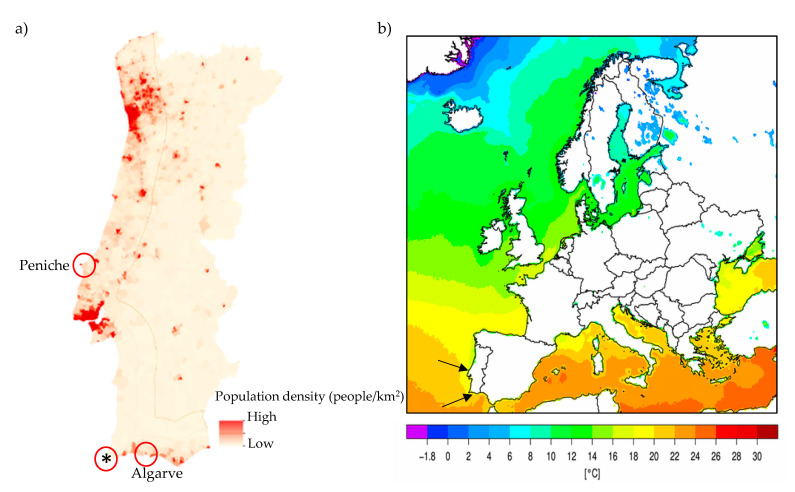
Localization of the fish sampling regions studied. (**a**) Population density on Portuguese coast (adapted) [16]; * indicates aquaculture sampling site. (**b**) Sea surface temperature during the summer of 2017 in Europe. Black arrows indicate the sampling sites on the Portuguese coast (adapted) [17].

Fish from two distinct regions of Portugal with different population density and ocean temperature, were analyzed during this study (Figure 1). A total of 15 gilthead seabreams caught along the coast of Peniche (*n* = 5, west/central Atlantic coast of Portugal), Algarve (*n* = 5, Portuguese southern Atlantic coast) and from an aquaculture farm in Algarve (*n* = 5) were purchased *post-mortem* from the respective commercial fisheries (Table 1). Additionally, 20 Atlantic horse mackerels were analyzed *post-mortem* from the same fisheries (Peniche, *n* = 10; Algarve, *n* = 10) (Table 1).

Fish have been divided by size and species. Larger fish, namely gilthead seabreams were gathered in pools of five specimens, while the smaller fish, Atlantic horse mackerel, in pools of 10. One internal tissue (liver—detoxification tissue and local of viral replication), and two external tissues (skin—contact tissue exposed to several environmental contaminants, and gills—portal of entry of many viral pathogens) were selected considering their characteristics. Approximately 2.0 g of tissue were chopped individually by using a sterilized razor blade to minimize external contamination. The dissection material was decontaminated with a solution of bleach a 10% and sterilized water between each sample manipulation. The tissues were homogenized in 10 mL of TNE buffer (50 mM Tris-HCl, 100 mM NaCl, 0.1 mM EDTA, pH 7.6) using a Precellys Evolution Homogenizer (Bertin Instruments, France). The homogenates previously obtained were centrifuged 10 min at 2000 rpm at 4 °C to remove any potential tissue debris, and the supernatant of each homogenized tissue was divided in triplicates and used for nucleic acids extraction. From the 45 samples, nucleic acids extraction was carried out from 250 µL of clarified supernatant, using NZYol (NZYTech, Portugal), following the manufacturer’s instructions. Nucleic acids were dissolved in 30 µL of DEPC-water, and the concentration and purity, of the obtained extracts determined using a NanoDrop 1000 spectrophotometer. The extracted nucleic acids were stored at −80 °C until further use.

### 2.2. Library Preparation and Sequencing

For all samples, libraries were prepared using the protocol described by Fernandez-Cassi et al. [18]. Briefly, in order to detect both RNA and DNA viruses, total RNA was retrotranscribed into cDNA using Invitrogen Superscript IV (Life technologies, Austin, TX, USA) and primer A, which is composed of 17-nucleotide specific sequence followed by a random nonamer for random priming A-(5′-GTTTCCCAGTCACGATANNNNNNNNN-3′) [19]. A cDNA complementary strand was synthesized using Sequenase 2.0 (USB/Affymetrix, Cleveland, OH, USA). To obtain enough DNA for sequencing library preparation, a pre-amplification PCR was performed using primer B (5′-GTTTCCCAGTCACGATA-3′) and AmpliTaqGold (Life technologies, Austin, Texas, USA). The temperature profile for this reaction was: 10 min at 95 °C as hot start, followed by 30 cycles of 30 s at 94 °C for denaturation, 30 s at 50 °C for annealing, and 1 min at 72 °C for extension. Finally, an extension at 72 °C for 10 min was used. To remove excess primers and dNTPs, PCR products were cleaned and concentrated using the Zymo DNA Clean and Concentrator kit (Zymo Research, USA). The concentration of the obtained DNA samples was determined using Qubit 2.0 (Life Technologies, USA), and for each sample, libraries were constructed using the KAPA HyperPlus Library Preparation kit (Roche, Switzerland) according to the manufacturer’s instructions.

Libraries were captured using VirCapSeq-VERT capture Panel (Roche, Switzerland). This panel consists of approximately two million probes, covering the genomes of 207 viral *taxa* known to infect vertebrates, thus enabling the detection of viral sequences in complex samples [20]. The samples prepared with the HyperCap Target Enrichment Kit (Roche, Switzerland) and the HyperCap Bead Kit (Roche, Switzerland), were hybridized with the VirCapSeq-VERT probes at 47 °C for 20 h. Immediately after the hybridization, the captured DNA samples were recovered with the Capture Beads (HyperCap Bead Kit, Roche) using a magnetic particle collector, and cleaned. The captured DNA, still bead-bounded, was purified using an ligation mediated PCR (LM-PCR). This post-capture PCR was purified and then sequenced in two runs using an Illumina MiSeq (2 × 300 bp) device, producing paired-end reads.

All reactions included non-template negative controls to rule out the possibility of positive amplification results due to external contamination.

### 2.3. Bioinformatic Pipeline

Bioinformatic processing and taxonomical assignment of the pair-end reads obtained as described above were analyzed using Genome Detective web-based software (https://www.genomedetective.com/) [21]. Briefly, low-quality reads were filtered and adapters trimmed using Trimmomatic [22]. Viral reads were selected using the DIAMOND protein-based alignment and non-viral sequences discarded. The obtained viral reads from Genome Detective were assembled with metaSPAdes [23] and taxonomically classified with NCBI-BLASTn and BLASTx tools to search for candidate reference sequences against the NCBI RefSeq virus database. In addition, assembled unused viral contigs and discovery viral contigs from Genome Detective software were analyzed using NCBI-BLASTn and tBLASTx tools against NCBI RefSeq viral databases using Python scripts. Only viral contigs with 85% identity and an e-value threshold below 10^−3^ (used as a cut-off) were used in this study.

### 2.4. Dataset Compilation and Phylogenetic Analysis

Nucleotide (nt) sequences used for the preparation of the different sequence-datasets were selected among those previously deposited in the GenBank database (Supplementary Materials Tables S1 and S2), on the proviso that they would be representative of (i) each of the previously described species with (ii) a significant sequence overlap with the sequences obtained in the course of this study, in order to maximize the number of unambiguously aligned nt positions in each sequence alignment. For phylogenetic analysis, multiple alignments of nt sequences were constructed with the iterative G-INS-I method as implemented in MAFFT vs. 7 [24] followed by their edition using GBlocks [25]. Phylogenetic trees were constructed using the Maximum Likelihood (ML) optimization criterium and the best fitting evolutionary model (GTR+Γ+I; GTR—general time reversal, Γ—Gamma distribution, I—proportion of invariant sites), as suggested W-IQ-TREE [26]. Phylogenetic reconstructions were carried out using either the Mega X software [27] run on a Linux server or W-IQ-TREE (version 2.1.2 MacOSX) [26] running on a personal computer, and the stability of the obtained ML tree topologies assessed by bootstrapping with 1000 re-samplings of the original sequence data. The sequence data files were deposited in GenBank within the BioProject with the SRA accession number PRJDB10531.

## 3. Results

### 3.1. Viral Sequences Diversity

#### 3.1.1. Viral Sequences Diversity—Global Results

We performed metagenomics analysis to characterize tissue-specific virome of two species of ray-finned (Actinopterygii) bony fish: the gilthead seabream and the Atlantic horse mackerel. This analysis was carried out using wild-caught specimens of gilthead seabreams and Atlantic horse mackerels, available from fisheries on the west (Peniche) and south (Algarve) Atlantic coast of Portugal, and farmed specimens of gilthead seabreams from the south (Algarve). To carry out this study, nucleic acids were extracted from the liver, gills, and skin of these animals, which was then pooled in libraries for next-generation sequencing. Overall, 1,216,428 viral reads were obtained that were assembled, *de novo*, into viral contigs for metagenomic analysis. 

**Figure 2:**
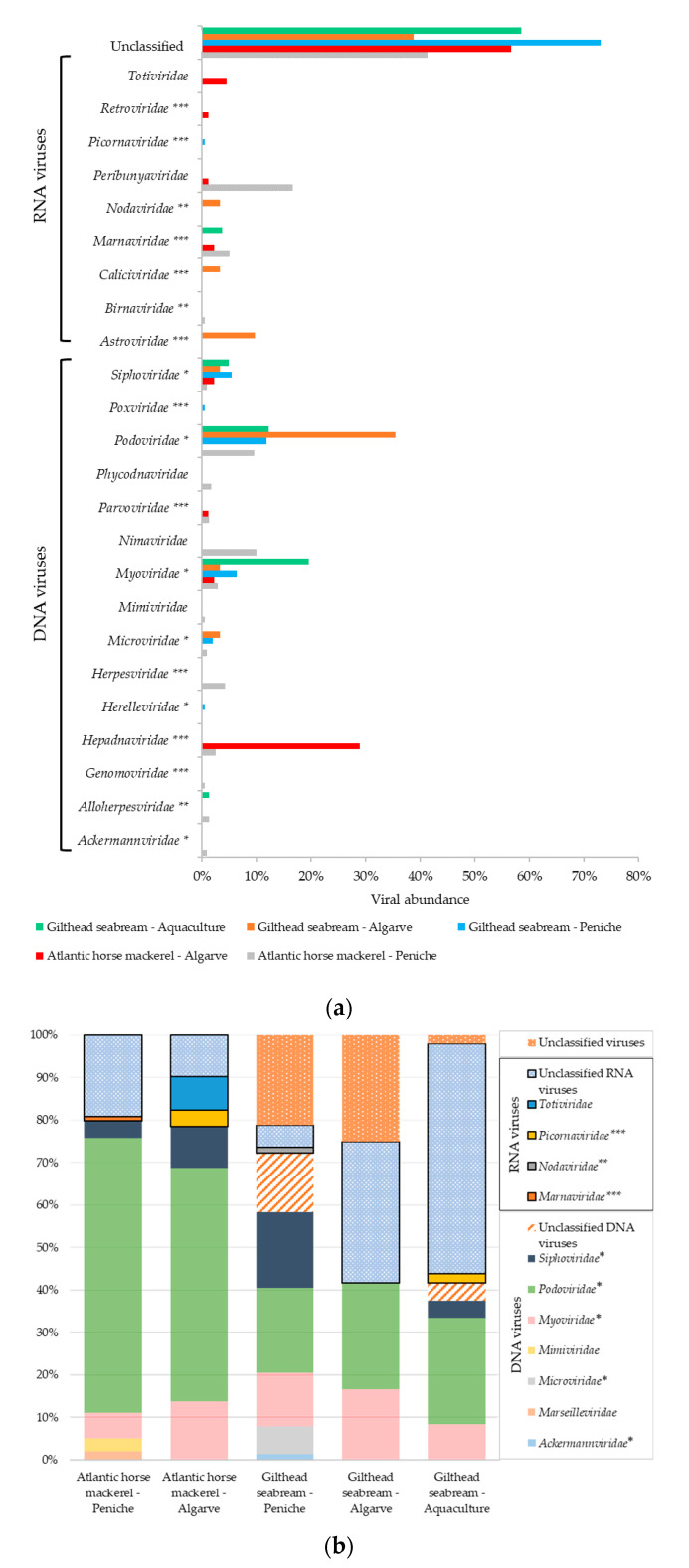
Blast results concerning viral diversity within each fish species and region. (**a**) Viral diversity in each fish species per region (including those contigs assigned as unclassified even within a family). (**b**) Viral diversity of unclassified viral contigs of each species per region. Bacteriophages families are pointed out with *, whereas pathogenic families for fish/other vertebrates and humans/other vertebrates are marked with ** and ***, respectively.

In total, the analysis revealed viral sequences that could be assigned to 25 different viral families (Figure 2). The analytical approach used in this study was able to identify both RNA and DNA viruses based on sequence similarity (Figure 2). The majority of the viral contigs were assigned to DNA viral families *Ackermannviridae*, *Alloherpesviridae*, *Genomoviridae*, *Hepadnaviridae*, *Herelleviridae*, *Herpesviridae*, *Marseilleviridae*, *Microviridae*, *Mimiviridae*, *Myoviridae*, *Nimaviridae*, *Parvoviridae*, *Phycodnaviridae*, *Podoviridae*, *Poxviridae*, and *Siphoviridae*. The remaining viral contigs were assigned to families of RNA viruses, including *Astroviridae*, *Birnaviridae*, *Caliciviridae*, *Marnaviridae*, *Nodaviridae*, *Picornaviridae*, *Peribunyaviridae*, *Retroviridae*, and *Totiviridae*. This analysis also revealed an unclassified taxonomic fraction of contigs (around 20%), with 3% of unclassified DNA viruses, 10% of unclassified RNA viruses, and 7% of viral sequences without any assignment to a known viral *taxon* after BLAST analysis [28,29].

#### 3.1.2. Atlantic Horse Mackerel and Gilthead Seabream Viral Sequence Diversity

**Figure 3:**
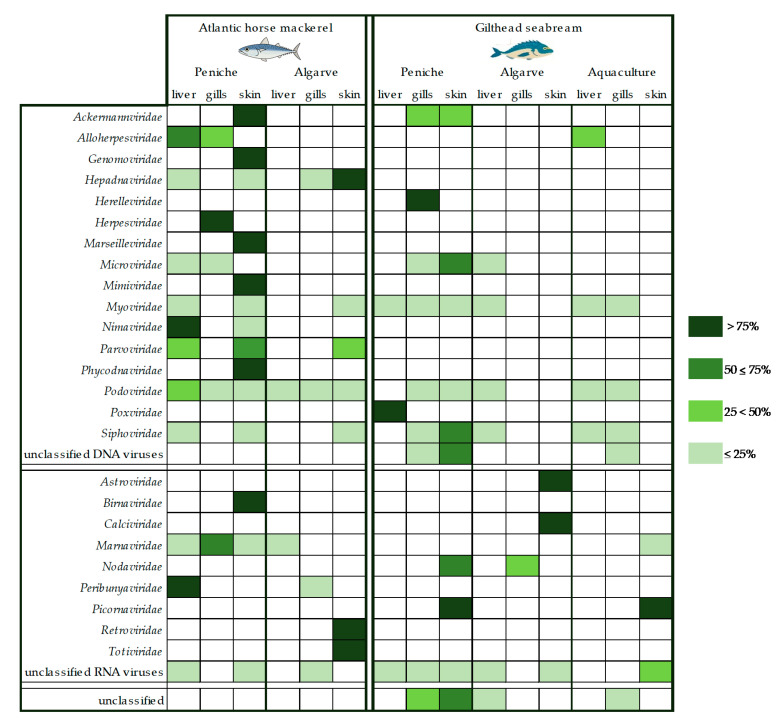
Viral abundance for each fish species, region, and tissue type normalized for each viral family (the taxonomic classification used is based on the Taxonomy Database from NCBI).

Our first analysis focused on parallel assessment of the viral sequence diversity between the two species selected, without considering their geographic or fishery/aquaculture origins. A comparison of viral abundance across the different studied organs for both species under analysis, revealed several differences. In total, viral sequences were more abundant in skin samples (44%) than in the liver (38%) or gills (18%). Bacteriophages from families *Myoviridae*, *Podoviridae*, and *Siphoviridae* were the viruses most frequently found in both fish species (Figure 2 and Figure 3). Podoviruses, which comprise short noncontractile-tailed bacteriophages with dsDNA genomes, were the most abundant (31%), totaling 19% and 8% of the total viral sequences associated with the liver and skin, respectively. The remaining 4% of podoviruses sequences were found in the gills. In the liver tissue samples prepared from Atlantic horse mackerel, the total virome associated with the *Podoviridae* family comprised 48% of the sequences analyzed, while the equivalent value for the gilthead seabream was only 14% (Figure 3). As for the gilthead seabream, we observed that almost 26% of the total virome for *Podoviridae* was associated with the skin tissues, whereas in the case of the Atlantic horse mackerel, the corresponding value was only 5%.

Additionally, the virome of mackerels from Peniche was apparently characterized by a higher, and more diverse number of viral sequences from different *taxa*, when compared with mackerels and seabreams from Algarve region, with viral sequences distributing among 17 viral families across the different organs (Figure 3). Although gilthead seabreams, in general, seemed to harbor a less diverse group of viral families regardless of the origin of the tissues under analysis, they displayed a higher abundance of unclassified viral sequences (Figure 2b and Figure 3).

Our data also revealed the presence of viral sequences from astroviruses, birnaviruses, caliciviruses, hepadnaviruses, nodaviruses, herpesviruses, and picornaviruses, all of which include representatives that are known to infect not only marine animals, such as fish and crustaceans, but also humans. For instance, *Birnaviridae*, *Hepadnaviridae*, and *Herpesviridae* viral sequences were identified in Atlantic horse mackerels, while others, indicating the presence of other families (*Astroviridae*, *Caliciviridae*, and *Nodaviridae*) were detected in libraries prepared from the skin and gills of gilthead seabreams.

#### 3.1.3. Viral Sequence Diversity: Fisheries vs. Aquaculture

An uneven distribution of viral diversity between fisheries from Peniche and Algarve, as well as from an aquaculture environment was observed. From the total of viral sequences obtained, the most abundant ones were associated with fish specimens from Peniche, with a total of 29% associated with the skin, followed by the liver (28%) and the gills (11%). The same pattern was observed in specimens from Algarve region, with 9%, 8%, and 2% of viral sequences being associated with the skin, liver, and gills, respectively. In aquaculture, 5% of viral sequences were detected in sequencing libraries prepared from skin samples, 3% from the liver and 5% from the gills.

As expected, and regardless the geographic origin of the specimens under analysis, bacteriophages were the most commonly found viruses, with *Podoviridae* being the most widespread family representing 21% and 7% of the total viral sequences detected in samples from Peniche and Algarve, respectively. In specimens from the Peniche region, we observed that 39% of the total liver virome was composed of podoviruses, although these could also be detected in libraries prepared from the skin (22%) and gills (7%). A similar trend was observed in specimens collected from Algarve, but the values associated with the liver (16%), the skin (4%), or the gills (2%) were lower.

It is notable that the Atlantic horse mackerel from Peniche, a more shoaling fish, had an associated higher number of distinct viral families (17 families) when compared to the gilthead seabream, a more solitary fish, with only 9 viral families identified (Figure 3). In contrast, Atlantic horse mackerels and gilthead seabreams from Algarve region presented a smaller number of viral families (9 and 7, respectively). Additionally, gilthead seabream from an aquaculture farm displayed the less diverse virome with our analysis, with nucleotide sequences allocated to only 6 viral families, half of which included bacteriophages. However, the unclassified taxonomic fraction associated with farmed gilthead seabream is substantial (56%).

### 3.2. Phylogenetic Relationships of Relevant Viral Pathogenic Families

**Figure 4:**
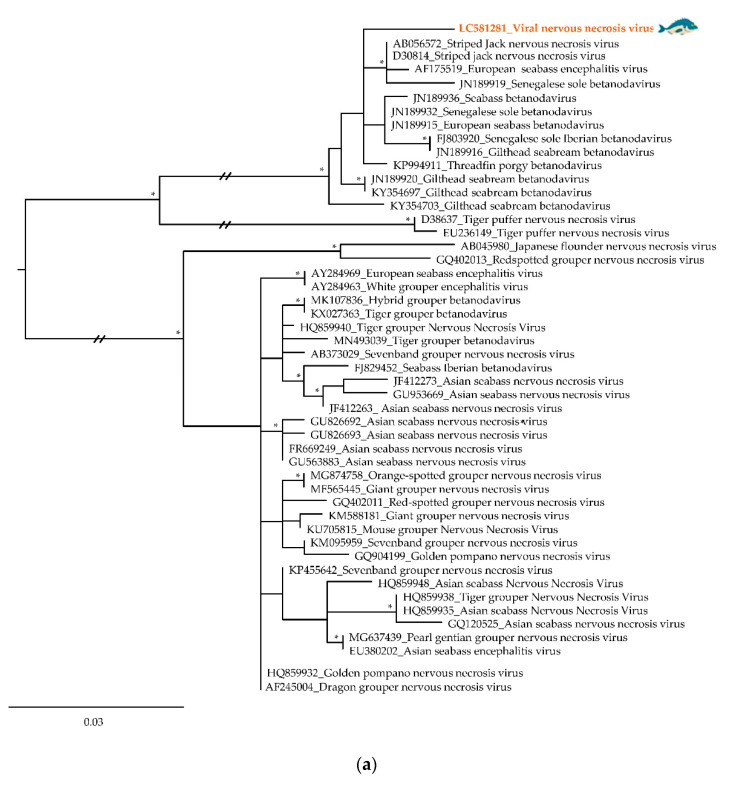

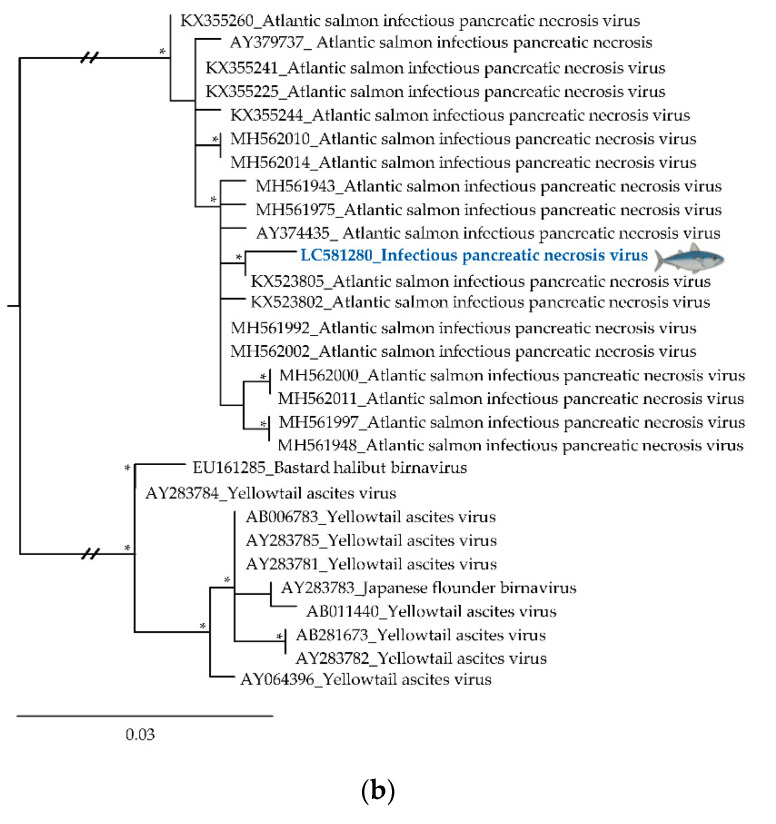

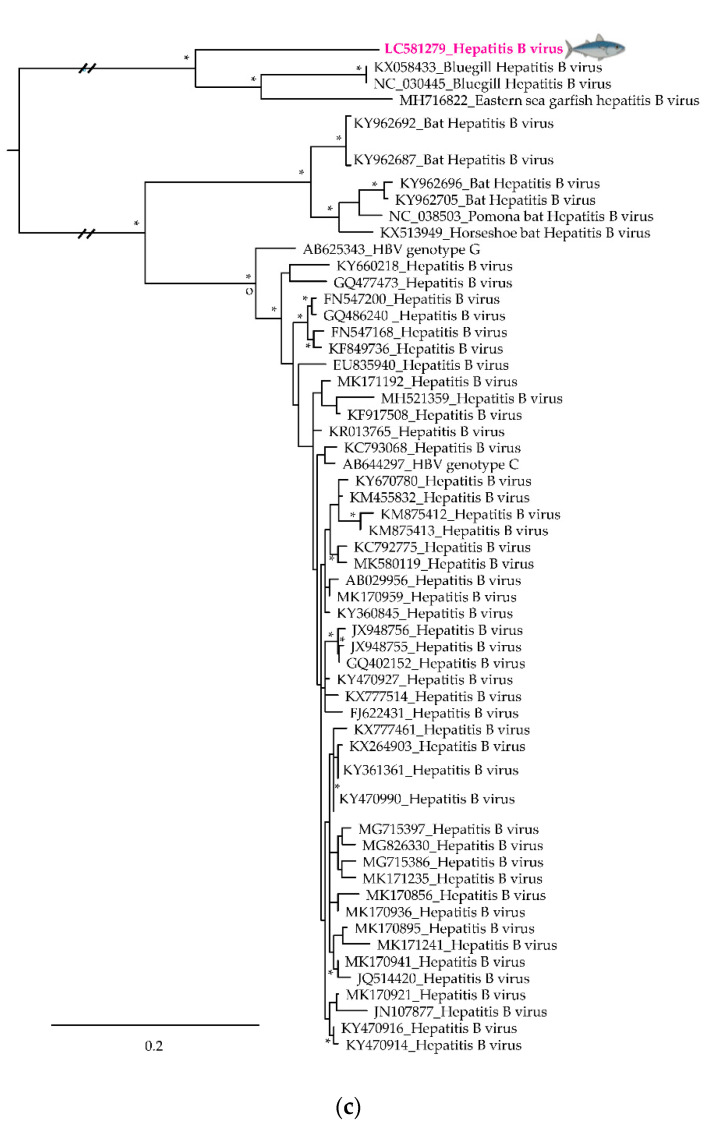
Phylogenetics trees of common fish pathogenic viruses: (**a**) *Nodaviridae*—partial sequence of Viral Nervous Necrosis Virus amplified from gilthead seabream gills from Algarve, (**b**) *Birnaviridae*—partial sequence of Infectious Pancreatic Necrotic Virus detected in Atlantic horse mackerel skin from Peniche, (**c**) *Hepadnaviridae*—partial sequence of Hepatitis B virus obtained from Atlantic horse mackerel gills from Algarve. The viral sequences analyzed in this study are colored and highlighted by a fish. All viral sequences are identified by their accession number and name. At specific branch nodes, bootstrap branches ≥0.60 are displayed by *; branch node point out with a circle referred to human viral sequences.

**Figure 5:**
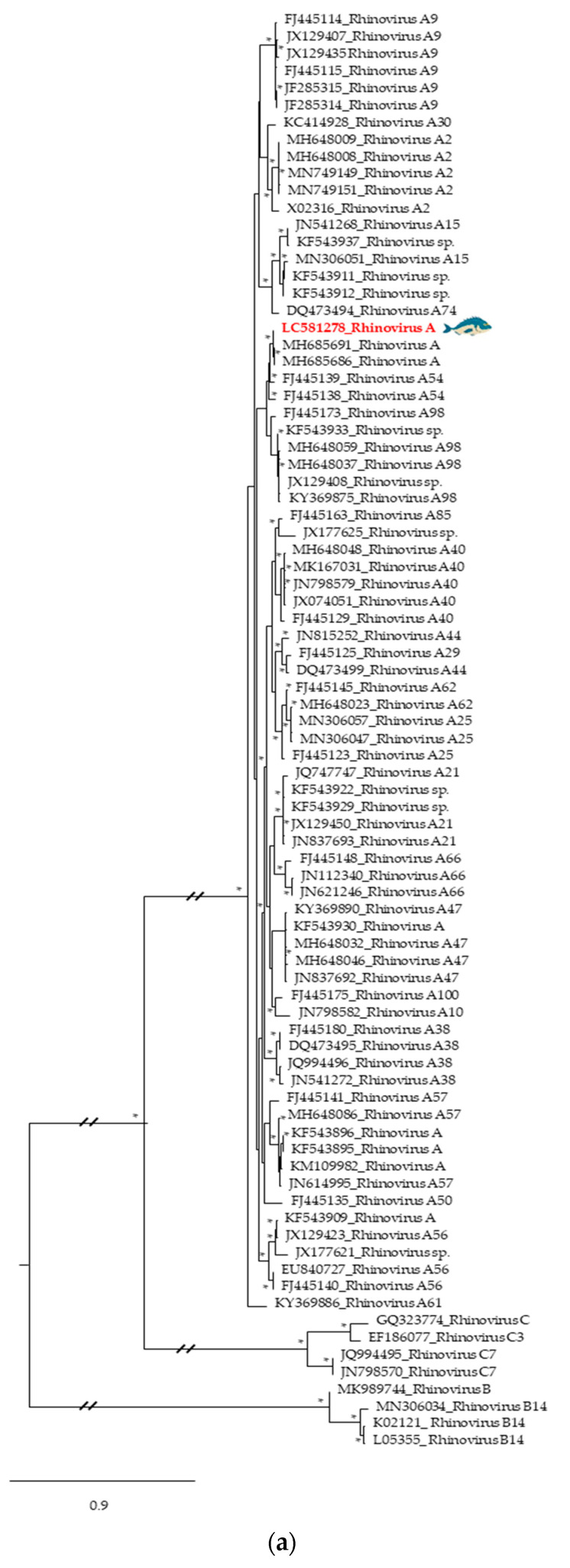

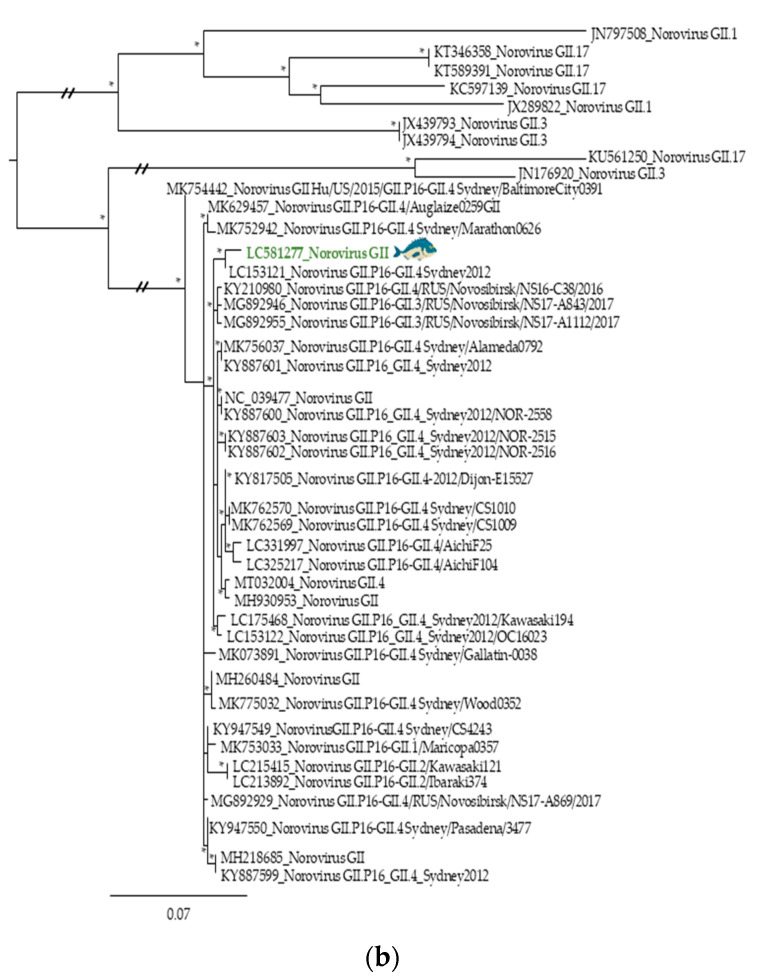
Phylogenetics trees of human pathogenic viruses identified in this study: (**a**) *Picornaviridae*—partial sequence of Rhinovirus A obtained from gilthead seabream skin from Peniche; (**b**) *Caliciviridae*—partial sequence of Norovirus GII amplified from gilthead seabream skin from Algarve. The viral sequences analyzed in this study are colored and highlighted by a blue fish. All viral sequences are identified by their accession number and name; all viral sequences used are from human hosts. At specific branch nodes bootstrap values ≥0.60 are displayed by *.

For further characterization of some relevant viral sequences from known human and fish pathogenic viruses from selected viral families, a phylogenetic analysis was carried out using the ML optimization criterium, and different sequence-datasets. The inferred phylogenetic trees (Figure 4 and Figure 5; Supplementary Materials Figures S1 and S2) revealed the phylogenetic relationships between the obtained sequences and their closest relatives, identified by sequence similarity searches at GenBank. All trees have been rooted at mid-point. 

Among the viral sequences identified, Viral Nervous Necrosis Virus (VNNV, accession number LC581281), one of upmost importance pathogenic nodaviruses to fish, which was detected in gilthead seabream from the Algarve, was placed, as expected, in a cluster with other betanodaviruses but branched separately from the other sequences previously identified (Figure 4a and Supplementary Materials Figure S1a). This group also included other viral reference sequences, previously detected in specimens of striped jack, European seabass, Senegalese sole, and gilthead seabream from Europe and Asia.

A birnavirus sequence (accession number LC581280), detected in Atlantic horse mackerel from Peniche (Figure 4b; Supplementary Materials Figure S1b), was placed, as expected from BLAST search results, with other Infectious Pancreatic Necrosis Viruses (IPNV) amplified from salmonids. Specifically, the closest homolog to the Atlantic horse mackerel IPNV sequence identified in this study corresponded to a putative virus detected in an Atlantic salmon (*Salmo salar*) caught in Chile. These sequences encoded putative proteins sharing 99.7% of amino acid identity. Interestingly, hepadnaviruses (which include Hepatitis B viruses (HBV) from various sources) were also identified from the gills of Atlantic horse mackerel but in this case from Algarve (accession number LC581279; Figure 4c and Supplementary Materials Figure S1c). The Atlantic horse mackerel HBV sequence here described branched separately from all the others in the dataset but was clearly closely related with other hepadnaviruses from fish, specifically, blue gill HBV, forming a separated cluster from the HBV that infect mammals. Additionally, a sequence amplified from the skin of gilthead seabream (accession number LC581278), clustered within a large monophyletic cluster among Rhinovirus A viral sequences from diverse sources, including those identified in humans, and clearly segregated away from the genetic lineages that include Rhinovirus B and Rhinovirus C sequences (Figure 5a and Supplementary Materials Figure S2a). The sequence detected shared 99% similarity with Rhinovirus A sequences detected in humans from Uganda (accession numbers MH685691 and MH685686). Another viral sequence obtained from gilthead seabream from Algarve (accession number LC581277) clustered in a major cluster grouping strains of NoV GII (Figure 5b and Supplementary Materials Figure S2b), specially NoV GII.P16-GII.4, phylogenetically close to the recombinant NoV GII.P16-GII.4 Sydney 2012 variant (Figure 5b), one of the most common NoV strains associated with outbreaks of gastroenteritis worldwide. 

## 4. Discussion

Viruses can be found in every single environment on Earth, but their importance is more evident in oceans, where they are known to be the reservoir of most of the genetic variety [5]. To explore the viral diversity of two important commercialized fish species, we analyzed viral sequences obtained from liver, gills, and skin tissue samples using a metagenomic approach. As these species included shoaling—Atlantic horse mackerel, and solitary—gilthead seabream specimens, the obtained data allowed us to tentatively address how fish population density might affect virus composition. 

The presence of unknown virus genomes in fish samples was expected since previous studies on aquatic viral communities have shown that the unknown (orphan) viral fraction (i.e., without homology to any sequence available in the databases) could be substantial, ranging between 20% to 99% [30,31]. In fact, the analysis of virus open reading frames (ORFs) showed that a large portion were orphans, with these unclassified ORFs being more frequently identified in viruses than in bacteria (30% and 9%, respectively) [32]. 

In addition, nucleotide sequences identifying viral families associated with vertebrate infections such as *Astroviridae*, *Nodaviridae*, *Hepadnaviridae*, *Birnaviridae*, *Caliciviridae*, and *Picornaviridae* were detected during this analysis (Figure 2 and Figure 3). Although, astroviruses have only recently been discovered in fish [7], they are known to persist in aquatic environments and have been detected in seabirds [33], amphibians [7], and mammals [34], where these viruses are a frequent cause of intestinal diseases [34]. Nodaviruses can cause neural necrosis, encephalopathy, or retinopathy in fish, being associated with behavioral abnormalities and high mortality, posing significant problems to marine aquaculture [35]. The analysis of a VNNV sequence detected in gilthead seabream gills, revealed that it was closely related to Striped jack nervous necrosis virus, the type species of the betanodaviruses. While, our sequence forms a separate branch with other betanodaviruses (Figure 4a), also detected in gilthead seabream, it segregates in a larger group of viral sequences that include others found in common fish species such as European seabass and Senegalese sole [36]. Interestingly, VNNV is endemic in the Mediterranean Sea, with several reports in fish samples [37,38,39], being in direct communication with the Atlantic Ocean through the Strait of Gibraltar, located at the south of Spain near the Algarve region, where part of this study was focused. In addition, other nodaviruses were also identified, namely the *Macrobrachium rosenbergii* nodavirus or *Penaeus vannamei* nodavirus, both pathogens of freshwater crustaceans that pose a threat to food security, and cause significant economic losses in the aquaculture industries [40].

Until recently, the known host range of hepadnaviruses was limited to mammals and birds [8]. However, previous studies showed that fish carry a remarkable diversity of hepadnaviruses [3,8]. Notably, the Atlantic horse mackerel Hepatitis B virus forms a sister group with other hepadnaviruses recently discovered in fish [8], segregating away from mammalian hepadnaviruses (Figure 4c and Supplementary Materials Figure S1c) which are known to cause infections that have the potential to lead to both severe chronic liver disease and hepatocellular carcinoma in humans [8].

IPNV is an important fish pathogen and the protype virus of the *Birnaviridae* family [41]. It has a worldwide distribution, and normally is associated with an acute and contagious disease that causes distended abdomen, aberrant swimming, darkened pigmentation, and necrotic lesions in internal organs. In this study, an IPNV sequence was detected in Atlantic horse mackerel skin tissue, corresponding to a distinct branch on a phylogenetic tree, separating it from other aquabirnaviruses such as the Yellowtail ascites virus (Figure 4b), the causative agent of ascites and responsible for serious losses in the fish-farming industry in Japan [42]. This sequence forms a group with IPNV sequences amplified from those clustering with others from Europe and South America specimens of salmonids [43]. This result is not surprising taking into account a report that supports the association between fish eggs from Europe and the disease transmission across Chilean farms [44].

Human NoV genomic sequence was also identified in the skin of gilthead seabream from the Algarve. Although, its presence does not mean that the virus is active or infectious, we cannot exclude the hypothesis that this virus can also be present in the fish flesh that can be consumed raw or undercooked in several meals, possessing a potential risk to human health. Noroviruses are the leading cause of viral gastroenteritis worldwide, and are estimated to cause 677 million cases and ≈ 210,000 human deaths every year [45].

Furthermore, the detection of NoV genomes in these fish samples can indicate that the water was contaminated with human feces, probably due to sewage pollution, or that fish were contaminated during handling, processing, or preparation. While the small size of the sequence under study limits the breath of observations drawn from its analysis, its place in a phylogenetic tree revealed that it branched with a group containing essentially recombinant strains of NoV GII.4 (Figure 5b) which caused six food-related outbreaks since 1995 [46]. In particular, the NoV sequence identified in this study is closely related to the recombinant GII.P16/GII.4 Sydney 2012. Interestingly, in the last decades, the GII.4 variant dominated NoV infections, being responsible for 60–80% of all NoV outbreaks. Therefore, in the last years, we observed the emergence of a novel GII.4 recombinant virus responsible for several outbreaks worldwide [45,46] that retained the Sydney 2012 capsid-coding sequence but acquired a new structural region (GII.P16/GII.4 Sydney 2012) [45,46]. 

Picornaviruses such as rhinoviruses, or other small non-enveloped viruses with RNA genomes such as noroviruses (*Caliciviridae*), have a compact capsid structure that has been selected to allow them to withstand a long-stand preservation in harsh environments [3]. A closer analysis of the rhinovirus sequence amplified from a sample of gilthead seabream skin from Peniche showed that this viral sequence branched separately from Rhinovirus B and C (Figure 5a). It formed a cluster mainly with Rhinovirus A serotypes 54 and 98, being phylogenetically close to rhinovirus sequences identified from stools in African children with diarrhea [47]. Although often ignored, human rhinoviruses are the most frequent causes of respiratory tract infections with severe disease manifestations in patients suffering from bronchiolitis and asthma [48]. 

Highly abundant viral sequences associated with the fish species studied were assigned to bacteriophage families, which are known as the most abundant viruses in the biosphere, occurring in large numbers in freshwater, soil, sewage, and marine environments [4]. Their presence in the environment suggests that they play an important role in the ecology and evolution of diverse ecosystems [5]. In particular, several bacteriophages from freshwater and marine ecosystems seem to play important roles not only in the equilibrium of the ecosystems as predators of bacteria, but also in a broad range of ecological processes such as carbon cycle [5,6]. 

The genomes of myoviruses, podoviruses, and shipoviruses were identified as the most widespread viral sequences found among the samples analyzed during this study. As expected, they were frequently associated with exterior organs (the skin), or tissues that were exposed to the external environment (the gills). Surprisingly, our data also suggest their presence in the liver. Considering that internal and external portions of the livers were used, it is likely that the majority of bacteriophage sequences detected in our samples would correspond to viruses found on the liver surface, in the abdominal cavity. Gut microbiota consists of a plethora of microorganisms including bacteria, fungi, and archaea, and a myriad of viruses including bacteriophages and others infecting eukaryotic cells. Bacteriophages have been found in humans and other animal, inhabiting the oral, gastrointestinal and respiratory tracts, in the urine and blood, the latter being considered an sterile environment in heathy individuals [49,50]. Moreover, the liver is an important component of the reticuloendothelial system [51], being one of the organs that filter a variety of foreign elements that are in circulation, including phages [51], being identified as one of the sites with the highest phage accumulation [30,31,52]. 

It has previously been shown that species of fish forming large and dense groups are characterized by high-level contact rates and exhibit higher viral diversity compared to their more solitary counterparts [3,53,54,55]. This hypothesis supports classic epidemiological theories that larger populations with higher contact rates have an increased likelihood of acquiring, and transmitting, viruses [56,57]. Interestingly, in our study, this only seems valid for the specimens caught in the region of Peniche since Atlantic horse mackerels and gilthead seabreams from Algarve fisheries do not reveal this pattern. These differences in the distribution of the viral families between the two fish populations could be due to sampling bias, differences in water temperature (around to 5 °C to 10 °C lower in Peniche than in the Algarve), anthropogenic factors, and recycled water management systems [57]. Thus, this hypothesis requires further studies to be investigated. Taking this into account, our data regarding the virome associated with liver and gills suggests that the most solitary fishes studied, the gilthead seabream from Peniche, houses the smallest viral diversity. On the other hand, Atlantic horse mackerel from the same region, a densely shoaling fish, held the greatest number of viral families across the studied organs. For this analysis, the skin tissue was not considered since it is an external contact organ more propitious to environmental and handling contaminations. Additionally, as expected, farmed gilthead seabream had the lower viral diversity, indicating more uniform rearing conditions [58]. Therefore, these results support, to some extent, the concept that more frequent intra-host contacts could increase viral diversification and spread. However, a broader comparison of more fish species is essential to understand how population density may influence virus diversity, abundance, and evolution [3].

Although this study focused on only two fish species commonly consumed in Europe, a characterization of their virome in different environments was made for the first time. Since the species analyzed were bought at fisheries, it is possible that we missed viruses with low abundance and gained other viruses (including phages) due to sample processing, manipulation, external contamination, and bacterial growth during fish transportation. Our findings support that fish harbor a very large number of viruses and that viral metagenomics is a useful tool to exhaustively characterize their viral-associated diversity. Indeed, metagenomic analysis has become a precious tool for the characterization of viral composition and diversity in environmental samples. However, the procedures used for sample collection, sequencing preparation and bioinformatic analyses may induce biases in estimating viral diversity [14]. Nevertheless, our knowledge, regarding the diversity of viruses in fish, has increased greatly with the development of metagenomic approaches [14]. Moreover, these approaches would be a valuable asset if used to understand the viral diversity and putative viral pathogens in aquaculture settings, since such information may directly impact on the biosafety of aquaculture systems [59]. Finally, the data presented here show that fish commonly sold in the markets, many of which may be consumed raw or undercooked, harbor a wide range of viral genomes, much of them, unclassified. This fact does not mean that the identified viruses are infectious or pathogenic, nor does it indicate their degree of virulence. However, we cannot exclude that at least some of these viruses can pose a potential risk to human health, being some of them human viral pathogens of interest regarding food safety. 

## Figures and Tables

**Table 1:** Fish samples acquired during this study, their source, and fishery type.

Species	Source	Fishery Type	Nº of Specimens
*Sparus aurata* (gilthead seabream)	Peniche fish market	Wild fisheries	5
*Sparus aurata* (gilthead seabream)	Algarve fish market	Wild fisheries	5
*Sparus aurata* (gilthead seabream)	Algarve fish market	Aquaculture	5
*Trachurus trachurus* (Atlantic horse mackerel)	Peniche fish market	Wild fisheries	10
*Trachurus trachurus* (Atlantic horse mackerel)	Algarve fish market	Wild fisheries	10

## Supplementary Materials

The following are available online at https://www.mdpi.com/2304-8158/9/11/1634/s1, Figure S1: Maximum Likelihood phylogenetics trees of each fish pathogenic viral family identified in this study, Figure S2: Maximum Likelihood phylogenetics trees of each human pathogenic viral family identified in this study, Table S1: Accession numbers of sequences used for phylogenetic analysis, including the sequences obtained in this study (*) and those downloaded from GenBank, Table S2: Accession numbers of sequences used for phylogenetic analysis (family analysis), including the sequences obtained in this study (*) and those downloaded from GenBank.
