# Supplementary material for: The Unexplored Virome of Two Atlantic Coast Fish: Contribution of Next-Generation Sequencing to Fish Virology
Source: Foods. 2020 Nov 9;9(11):1634. doi: 10.3390/foods9111634 (PMC7695296; doi:10.3390/foods9111634)
Supplement: Supplementary file 1 [file foods-09-01634-s001.pdf]

Table S1. Accession numbers of sequences used for phylogenetic analysis, including the sequences obtained in this study (*) and those downloaded from GenBank: a) sequences used for phylogenetic analysis of Viral Nervous Necrosis Virus obtained from a gilthead seabream gills from Algarve, b) sequences used for phylogenetic analysis of Infectious Pancreatic Necrotic Virus amplified from an Atlantic horse mackerel skin sample from Peniche, c) sequences used for phylogenetic analysis of Hepatitis B virus obtained from Atlantic horse mackerel gills from Algarve, d) sequences used for phylogenetic analysis of Rhinovirus A amplified from gilthead seabream skin from Peniche; e) sequences used for phylogenetic analysis of Norovirus GII obtained from gilthead seabream skin from Algarve.

a)

Country	Origin	Access number
Japan	Striped jack	AB056572
Japan	Striped jack	D30814
Japan	European seabass	AF175519
Italy	European Seabass	JN189936
Spain	Senegalese sole	JN189919
Italy	Senegalese sole	JN189932
Croatia	European seabass	JN189915
Portugal	Senegalese sole	FJ803920
Portugal	Seabream	JN189916
China	Threadfin porgy	KP994911
Cyprus	Seabream	JN189920
Cyprus	Seabream	KY354697
Italy	Seabream	KY354703
Japan	Tiger puffer	D38637
Japan	Tiger puffer	EU236149
Japan	Japanese flounder	AB045980
Australia	Redspotted grouper	GQ402013
Israel	European Seabass	AY284969
Israel	White Grouper	AY284963
China	Hybrid grouper	MK107836
China	Tiger Grouper	KX027363
China	Tiger grouper	HQ859940
Malaysia	Tiger grouper	MN493039
Japan	Sevenband grouper	AB373029
Spain	Seabass	FJ829452
India	Asian seabass	JF412273
India	Asian seabass	GU953669
India	Asian seabass	JF412263
India	Asian seabass	GU826692
India	Asian seabass	GU826693
India	Asian seabass	FR669249
India	Asian seabass	GU563883
China	Orange-spotted grouper	MG874758
Taiwan	Giant grouper	MF565445
Australia	Redspotted grouper	GQ402011
Taiwan	Giant grouper	KM588181
Vietnam	Mouse grouper	KU705815
South Korea	Sevenband grouper	KM095959
Malaysia	Golden pompano	GQ904199
China	Sevenband grouper	KP455642
Malaysia	Asian seabass	HQ859948
Malaysia	Tiger grouper	HQ859938
Malaysia	Asian seabass	HQ859935
Malaysia	Asian seabass	GQ120525

China	Pearl gentian	MG637439
Malaysia	Asian seabass	EU380202
Malaysia	Golden pompano	HQ859932
Taiwan	Dragon grouper	AF245004
Portugal	Seabream	LC581281*

b)

Country	Origin	Access number
Norway	Atlantic salmon	KX355260
Norway	Atlantic salmon	AY379737
Norway	Atlantic salmon	KX355241
Norway	Atlantic salmon	KX355225
Norway	Atlantic salmon	KX355244
Norway	Atlantic salmon	MH562010
Norway	Atlantic salmon	MH562014
Norway	Atlantic salmon	MH561943
Norway	Atlantic salmon	MH561975
Norway	Atlantic salmon	AY374435
Chile	Atlantic salmon	KX523805
Chile	Atlantic salmon	KX523802
Norway	Atlantic salmon	MH561992
Norway	Atlantic salmon	MH562002
Norway	Atlantic salmon	MH562000
Norway	Atlantic salmon	MH562011
Norway	Atlantic salmon	MH561997
Norway	Atlantic salmon	MH561948
China	Japanese flounder/ Bastard halibut	EU161285
South Korea	Japanese flounder	AY283784
Japan	Yellowtail	AB006783
Japan	Japanese amberjack/ yellowtail	AY283785
Japan	Yellowtail	AY283781
Japan	Japanese flounder	AY283783
Japan	Yellowtail	AB011440
Japan	Yellowtail	AB281673
Japan	Yellowtail	AY283782
Korea	Rockfish	AY064396
Portugal	Atlantic horse mackerel	LC581280*

c)

Country	Origin	Access number
USA	Bluegill	KX058433
USA	Bluegill	NC_030445
Australia	Eastern sea garfish	MH716822
China	Greater horseshoe bat	KY962692
China	Greater horseshoe bat	KY962687
China	Woolly horseshoe bat	KY962696
China	Least horseshoe bat	KY962705
China	Pomona roundleaf bat	NC_038503
China	Horseshoe bat	KX513949
Mexico	Human	AB625343
Russia	Human	KY660218
Poland	Human	GQ477473
Nigeria	Human	FN547200

France	Human	GQ486240
Nigeria	Human	FN547168
Angola	Human	KF849736
Taiwan	Human	EU835940
China	Human	MK171192
Netherlands	Human	MH521359
China	Human	KF917508
China	Human	KR013765
Taiwan	Human	KC793068
Indonesia	Human	AB644297
China	Human	KY670780
China	Human	KM455832
China	Human	KM875412
China	Human	KM875413
Taiwan	Human	KC792775
China	Human	MK580119
Japan	Human	AB029956
China	Human	MK170959
China	Human	KY360845
China	Human	JX948756
China	Human	JX948755
China	Human	GQ402152
China	Human	KY470927
China	Human	KX777514
China	Human	FJ622431
China	Human	KX777461
South Korea	Human	KX264903
China	Human	KY361361
China	Human	KY470990
China	Human	MG715397
China	Human	MG826330
China	Human	MG715386
China	Human	MK171235
China	Human	MK170856
China	Human	MK170936
China	Human	MK170895
China	Human	MK171241
China	Human	MK170941
Bangladesh	Human	JQ514420
China	Human	MK170921
India	Human	JN107877
China	Human	KY470916
China	Human	KY470914
Portugal	Atlantic horse mackerel	LC581279*

d)

**Country**	**Origin**	**Access number**
USA	Human	FJ445114
El Salvador	Human	JX129407
Peru	Human	JX129435
USA	Human	FJ445115
Switzerland	Human	JF285315
Switzerland	Human	JF285314
South Korea	Human	KC414928
Singapore	Human	MH648009

Singapore	Human	MH648008
USA	Human	MN749149
USA	Human	MN749151
Austria	Human	X02316
USA	Human	JN541268
Australia	Human	KF543937
USA	Human	MN306051
Australia	Human	KF543911
Australia	Human	KF543912
USA	Human	DQ473494
Uganda	Human	MH685691
Uganda	Human	MH685686
USA	Human	FJ445139
USA	Human	FJ445138
USA	Human	FJ445173
Australia	Human	KF543933
Singapore	Human	MH648059
Singapore	Human	MH648037
El Salvador	Human	JX129408
USA	Human	KY369875
USA	Human	FJ445163
South Korea	Human	JX177625
Singapore	Human	MH648048
USA	Human	MK167031
USA	Human	JN798579
USA	Human	JX074051
USA	Human	FJ445129
USA	Human	JN815252
USA	Human	FJ445125
USA	Human	DQ473499
USA	Human	FJ445145
Singapore	Human	MH648023
USA	Human	MN306057
USA	Human	MN306047
USA	Human	FJ445123
USA	Human	JQ747747
Australia	Human	KF543922
Australia	Human	KF543929
Peru	Human	JX129450
USA	Human	JN837693
USA	Human	FJ445148
USA	Human	JN112340
USA	Human	JN621246
USA	Human	KY369890
Australia	Human	KF543930
Singapore	Human	MH648032
Singapore	Human	MH648046
USA	Human	JN837692
USA	Human	FJ445175
USA	Human	JN798582
USA	Human	FJ445180
USA	Human	DQ473495
USA	Human	JQ994496
USA	Human	JN541272
USA	Human	FJ445141

Singapore	Human	MH648086
Australia	Human	KF543896
Australia	Human	KF543895
India	Human	KM109982
USA	Human	JN614995
USA	Human	FJ445135
Australia	Human	KF543909
Columbia	Human	JX129423
South Korea	Human	JX177621
Switzerland	Human	EU840727
USA	Human	FJ445140
USA	Human	KY369886
Australia	Human	GQ323774
Australia	Human	EF186077
USA	Human	JQ994495
USA	Human	JN798570
Kenya	Human	MK989744
USA	Human	MN306034
USA	Human	K02121
USA	Human	L05355
Portugal	Seabream	LC581278*

e)

Country	Origin	Access number
USA	Human	JN797508
Hong Kong	Human	KT346358
Hong Kong	Human	KT589391
French Guiana	Human	KC597139
South Korea	Human	JX439793
South Korea	Human	JX439794
South Korea	Human	KU561250
Netherlands	Human	JN176920
USA	Human	MK754442
USA	Human	MK629457
USA	Human	MK752942
Japan	Human	LC153121
Russia	Human	MG892946
Russia	Human	MG892955
USA	Human	MK756037
United Kingdom	Human	KY887601
United Kingdom	Human	NC_039477
United Kingdom	Human	KY887600
United Kingdom	Human	KY887603
United Kingdom	Human	KY887602
United Kingdom	Human	KY817505
USA	Human	MK762570
USA	Human	MK762569
Japan	Human	LC331997
Japan	Human	LC325217
USA	Human	MT032004
Portugal	Human	MH930953
Japan	Human	LC175468
Japan	Human	LC153122
USA	Human	MK073891
USA	Human	MH260484

USA	Human	MK775032
USA	Human	KY947549
USA	Human	MK753033
Japan	Human	LC215415
Japan	Human	LC213892
Russia	Human	MG892929
USA	Human	KY947550
United Kingdom	Human	MH218685
United Kingdom	Human	KY887599
Portugal	Seabream	LC581277*

Table S2. Accession numbers of sequences used for phylogenetic analysis (family analysis), including the sequences obtained in this study (*) and those downloaded from GenBank: a) sequences used for phylogenetic analysis of Viral Nervous Necrosis Virus detected in gilthead seabream gills from Algarve, b) sequences used for phylogenetic analysis of Infectious Pancreatic Necrotic Virus amplified from Atlantic horse mackerel skin from Peniche, c) sequences used for phylogenetic analysis of Hepatitis B virus obtained from Atlantic horse mackerel gills from Algarve, d) sequences used for phylogenetic analysis of Rhinovirus A detected in a gilthead seabream skin sample from Peniche; e) sequences used for phylogenetic analysis of Norovirus GII obtained from gilthead seabream skin from Algarve.

a)

Country	Origin	Access number
Japan	Barfin flounder	NC_013459
Japan	Barfin flounder	EU826138
Japan	Redspotted grouper	NC_008041
China	Redspotted grouper	AY690596
Japan	Tiger puffer	NC_013461
Japan	Tiger puffer	EU236149
Portugal	Gilthead seabream	LC581281*
Japan	Striped jack	NC_003449
Japan	Striped jack	D30814
USA	Mosquitoes	NC_004145
USA	Mosquitoes	X15960
USA	Mosquitoes	NC_004144
USA	Black beetle	X15959
USA	Black beetle	X00956
USA	Mosquitoes	NC_002691
USA	Mosquitoes	AF174534
Peru	Southern armyworm	NC_003692
Peru	Southern armyworm	AF171943

b)

Country	Origin	Access number
Germany	Chicken	X84034
France	Chicken	AJ310185
Canada	Insects	NC_004177
China	Mosquitoes	JX403941
Germany	Mosquitoes	JQ659254
France	Rotifer	FM995220
USA	Mosquitoes	GQ342962
Singapore	Asian seabass	MK103419
Vietnam	Blotched snakehead	NC_005982
Vietnam	Blotched snakehead	AJ459382
Japan	Yellowtail	NC_004168

Japan	Yellowtail	AY283781
Canada	Fish	NC_001915
Canada	Fish	M18049
Portugal	Atlantic horse mackerel	LC581280*
USA	Bivalve	AF342731
United Kingdom	Bivalve	AJ920336
United Kingdom	Bivalve	NC_038870

c)

Country	Origin	Access number
Brazil	Capuchin monkey	KY703886
Brazil	Capuchin monkey	NC_043528
USA	Woolly monkey	AF046996
USA	Woolly monkey	NC_028129
USA	Human	NC_003977
USA	Human	X02763
China	White-toothed shrew	MH484442
China	Asian gray shrew	MH484438
Myanmar	Bat	NC_020881
China	Pomona roundleaf bat	KF939649
China	Pomona roundleaf bat	NC_038503
Gabon	Noack's roundleaf bat	NC_024443
Gabon	Noack's roundleaf bat	KC790376
Ivory Coast	Antelope	MK620908
USA	Ground squirrel	NC_001484
USA	Ground squirrel	K02715
USA	Woodchuck	M19183
USA	Woodchuck	NC_004107
Australia	Domestic cat	MH307930
Australia	Domestic cat	NC_040719
Panama	Tent making bat	KC790381
Panama	Tent making bat	NC_024445
Portugal	Atlantic horse mackerel	LC581279 *
USA	Bluegill	KX058433
USA	Bluegill	NC_030445
USA	White sucker	NC_027922
USA	White sucker	KR229754
Germany	Heron	NC_001486
Germany	Heron	M22056
Canada	Duck	AF047045
China	Duck	NC_001344
Poland	Parakeet	JX274035
Poland	Parakeet	NC_016561
China	Tibetan frog	MH700450
China	Tibetan frog	NC_030446

d)

Country	Origin	Access number
Finland	Human	NC_001612
Japan	Human	NC_001472
Russia	Human	AY896767
China	African green monkey	MN427525
China	African green monkey	MN427527
China	African green monkey	MN427528
China	Rhesus monkey	KU587555

China	Rhesus monkey	NC_029905
Nigeria	Wastewater	MT542698
Germany	Bovine	MT019688
Germany	Bovine	NC_021220
USA	Human	DQ092795
Dubai	Dromedary	NC_038310
Dubai	Dromedary	KP345888
Japan	Wild boar	LC316832
Japan	Wild boar	LC316831
Japan	Wild boar	LC316819
United Kingdom	Human	NC_001430
Cameroon	Human	MK032898
Malawi	Human	MN914205
China	Rodent	KX156158
China	Rodent	NC_038989
USA	Simian	NC_038309
USA	Simian	AF326759
USA	Human	JX193795
USA	Human	JX074053
Malaysia	Human	KJ675506
Hong Kong	Human	NC_009996
USA	Human	JX074055
Portugal	Gilthead seabream	LC581278*
USA	Environment	MF043119

e)

Country	Origin	Access number
Japan	Wild boar	AB863586
Canada	Wild boar	NC_012699
USA	Rhesus monkey	NC_043512
USA	Rhesus monkey	EU391643
France	Hare	NC_002615
Italy	Wolf	MF356366
Portugal	Rabbit	KF442964
China	Alpine Musk Deer	MN478485
United Kingdom	Cattle	DQ013304
United Kingdom	Cattle	NC_007916
United Kingdom	Feline	M86379
USA	Feline	NC_001481
USA	Pig	NC_002551
USA	Pig	U76874
Sweden	Human	KX431437
Sweden	Human	KY040366
Brazil	Chicken	NC_033081
Germany	Chicken	HQ010042
Germany	Turkey	NC_043516
Germany	Turkey	JQ347522
Germany	Human	AF093797
Portugal	Gilthead seabream	LC581277*
Vietnam	Human	AF504671
USA	Fathead minnow	KX371097
USA	Fathead minnow	NC_035675
Canada	Atlantic salmon	MN995807
Norway	Atlantic salmon	NC_024031

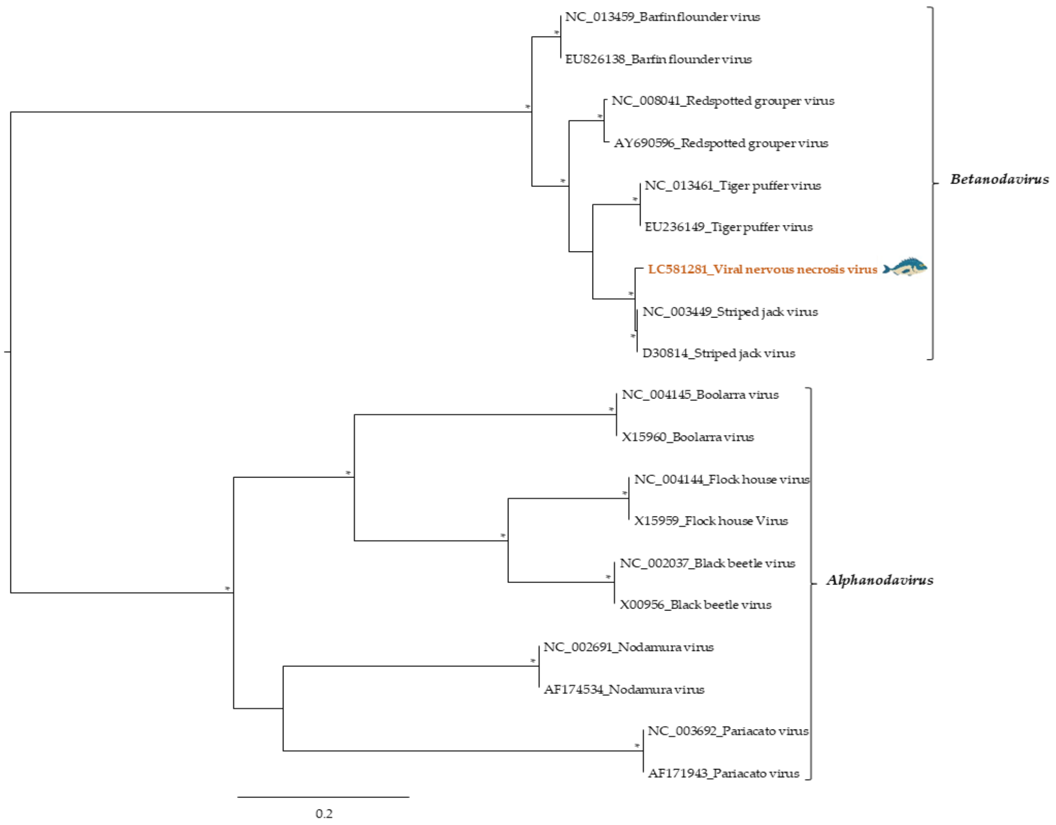

NC_013459_Barfin flounder virus
EU826138_Barfin flounder virus
NC_008041_Redspotted grouper virus
AY690596_Redspotted grouper virus
NC_013461_Tiger puffer virus
EU236149_Tiger puffer virus
LC581281_Viral nervous necrosis virus
NC_003449_Striped jack virus
D30814_Striped jack virus
Betanodavirus
NC_004145_Boolarra virus
X15960_Boolarra virus
NC_004144_Flock house virus
X15959_Flock house Virus
NC_002037_Black beetle virus
X00956_Black beetle virus
Alphanodavirus
NC_002691_Nodamura virus
AF174534_Nodamura virus
NC_003692_Pariacato virus
AF171943_Pariacato virus
0.2

(a)

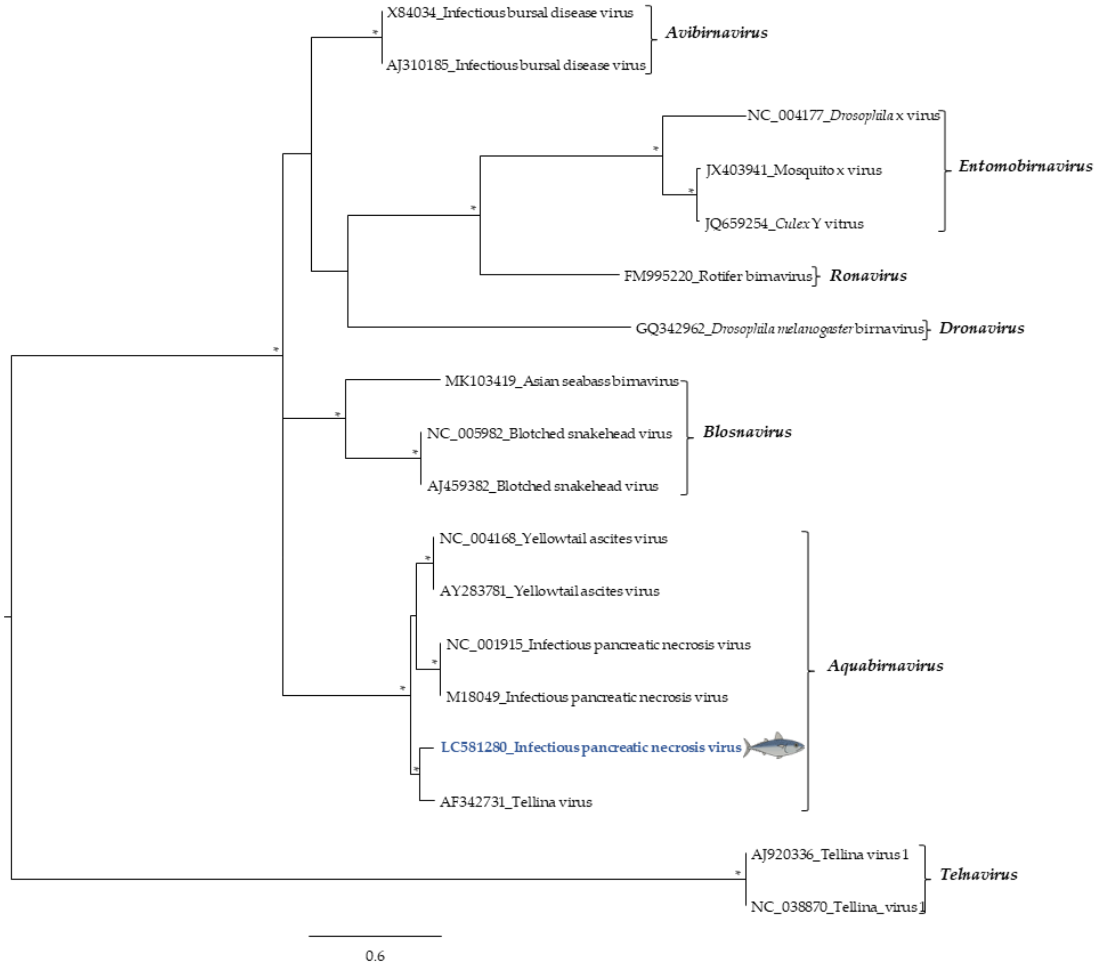
X84034_Infectious bursal disease virus
AJ310185_Infectious bursal disease virus
Avibirnavirus
NC_004177_Drosophila x virus
JX403941_Mosquito x virus
JQ659254_Culex Y vitrus
Entomobirnavirus
FM995220_Rotifer birnavirus
Ronavirus
GQ342962_Drosophila melanogaster birnavirus
Dronavirus
MK103419_Asian seabass birnavirus
NC_005982_Blotched snakehead virus
AJ459382_Blotched snakehead virus
Blosnavirus
NC_004168_Yellowtail ascites virus
AY283781_Yellowtail ascites virus
NC_001915_Infectious pancreatic necrosis virus
M18049_Infectious pancreatic necrosis virus
LC581280_Infectious pancreatic necrosis virus
AF342731_Tellina virus
Aquabirnavirus
AJ920336_Tellina virus 1
NC_038870_Tellina_virus1
Telnavirus
0.6

(b)

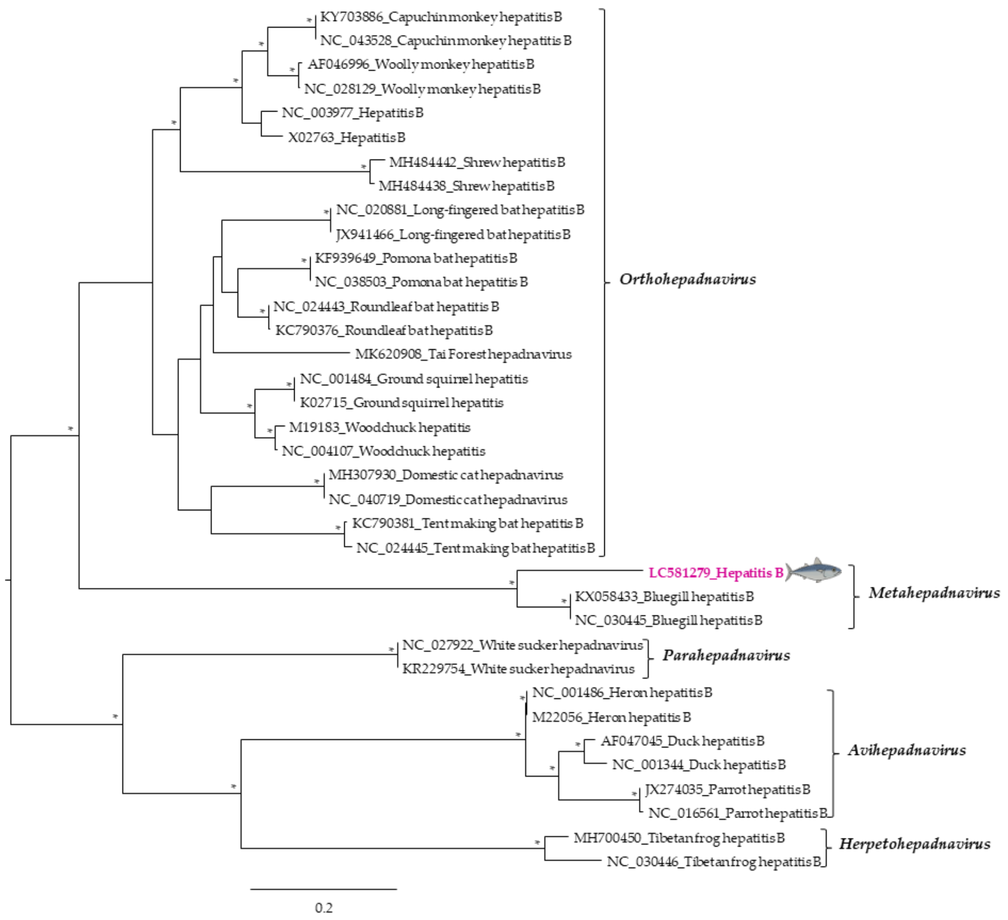

(c)

Figure S1. Maximum Likelihood phylogenetics trees of each fish pathogenic viral family identified in this study: a) *Nodaviridae* (including a partial sequence of Viral Nervous Necrosis Virus detected in gilthead seabream gills from Algarve), b) *Birnaviridae* (including a partial sequence of Infectious Pancreatic Necrotic Virus obtained from Atlantic horse mackerel skin from Peniche), and c) *Hepadnaviridae* (including a partial sequence of Hepatitis B virus obtained from Atlantic horse mackerel gills from Algarve). The viral sequences analysed in this study are coloured and highlighted by fish. All viral sequences are identified by their accession number and name. At specific branch nodes, bootstrap values ≥0.60 are displayed by *.

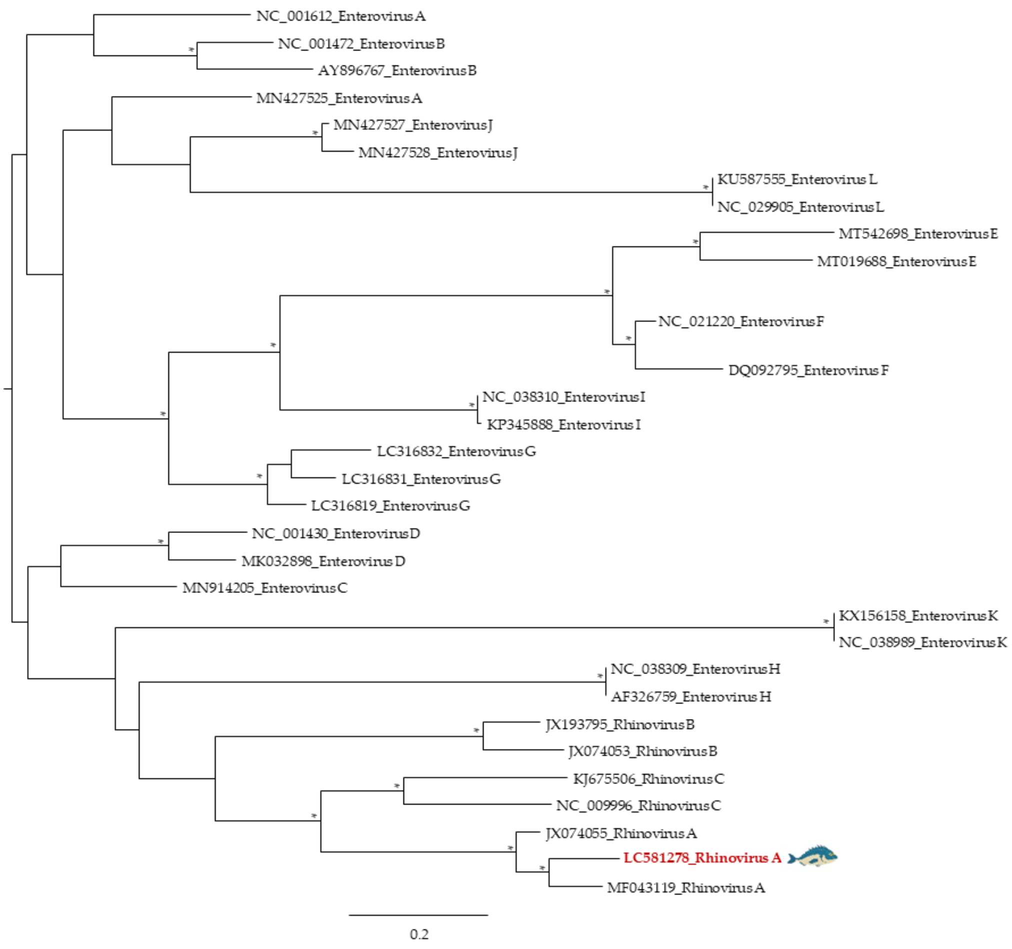
NC_001612_Enterovirus A
NC_001472_Enterovirus B
AY896767_Enterovirus B
MN427525_Enterovirus A
MN427527_Enterovirus J
MN427528_Enterovirus J
KU587555_Enterovirus L
NC_029905_Enterovirus L
MT542698_Enterovirus E
MT019688_Enterovirus E
NC_021220_Enterovirus F
DQ092795_Enterovirus F
NC_038310_Enterovirus I
KP345888_Enterovirus I
LC316832_Enterovirus G
LC316831_Enterovirus G
LC316819_Enterovirus G
NC_001430_Enterovirus D
MK032898_Enterovirus D
MN914205_Enterovirus C
KX156158_Enterovirus K
NC_038989_Enterovirus K
NC_038309_Enterovirus H
AF326759_Enterovirus H
JX193795_Rhinovirus B
JX074053_Rhinovirus B
KJ675506_Rhinovirus C
NC_009996_Rhinovirus C
JX074055_Rhinovirus A
LC581278_Rhinovirus A
MF043119_Rhinovirus A
0.2

(a)

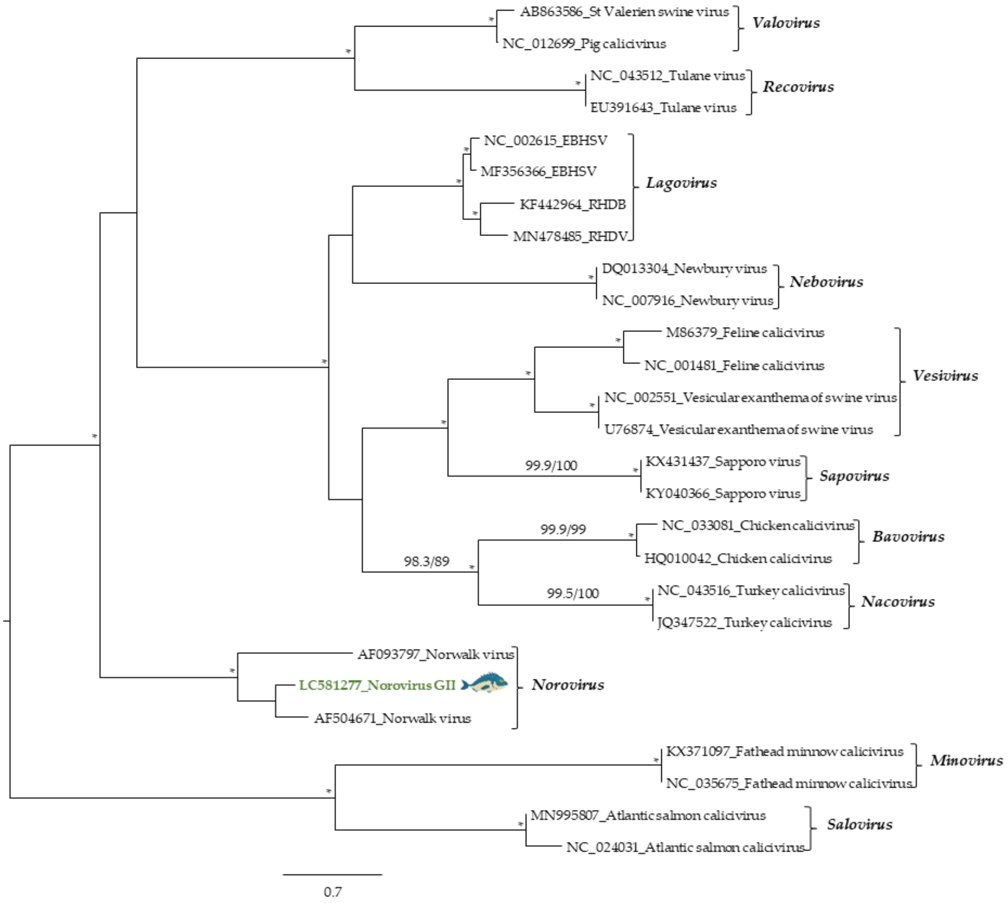

(b)

Figure S2. Maximum Likelihood phylogenetics trees of each human pathogenic viral family identified in this study: a) *Picornaviridae* (partial sequence of Rhinovirus A obtained in gilthead seabream skin from Peniche); b) *Caliciviridae* (partial sequence of Norovirus GII obtained in gilthead seabream skin from Algarve). Due to the hight genetic diversity observed in *Picornaviridae*, the phylogenetic tree for this taxon was constructed based on the alignment of the closest sequences from a phylogenetic point of view. The viral sequences analysed in this study are coloured and highlighted by a blue fish. All viral sequences are identified by their accession number and name. At specific branch nodes, bootstrap values ≥0.60 are displayed by *.
